# Variations in the Intragene Methylation Profiles Hallmark Induced Pluripotency

**DOI:** 10.1155/2015/976362

**Published:** 2015-11-05

**Authors:** Pavel Druzhkov, Nikolay Zolotykh, Iosif Meyerov, Ahmed Alsaedi, Maria Shutova, Mikhail Ivanchenko, Alexey Zaikin

**Affiliations:** ^1^Department of Algebra, Geometry and Discrete Mathematics, Lobachevsky State University of Nizhny Novgorod, Nizhny Novgorod, Russia; ^2^Department of Mathematical Software and High-Performance Computing, Lobachevsky State University of Nizhny Novgorod, Nizhny Novgorod, Russia; ^3^Department of Mathematics, King Abdulaziz University, Jeddah, Saudi Arabia; ^4^Stem Cell Laboratory, Vavilov Institute of General Genetics, RAS, Moscow, Russia; ^5^Department of Applied Mathematics and Center of Bioinformatics, Lobachevsky State University of Nizhny Novgorod, Nizhny Novgorod, Russia; ^6^Institute for Women's Health and Department of Mathematics, University College London, London, UK

## Abstract

We demonstrate the potential of differentiating embryonic and induced pluripotent stem cells by the regularized linear and decision tree machine learning classification algorithms, based on a number of intragene methylation measures. The resulting average accuracy of classification has been proven to be above 95%, which overcomes the earlier achievements. We propose a constructive and transparent method of feature selection based on classifier accuracy. Enrichment analysis reveals statistically meaningful presence of stemness group and cancer discriminating genes among the selected best classifying features. These findings stimulate the further research on the functional consequences of these differences in methylation patterns. The presented approach can be broadly used to discriminate the cells of different phenotype or in different state by their methylation profiles, identify groups of genes constituting multifeature classifiers, and assess enrichment of these groups by the sets of genes with a functionality of interest.

## 1. Introduction

The studies of embryonic stem cells (ESCs) and induced pluripotent stem cells (iPSCs) constitute a focal point of modern developmental biology and regenerative medicine [1–4]. Reprogramming somatic cells (SCs) to the pluripotent state presents both the fundamental interest of understanding and controlling the development and the practical importance of circumventing ethical and logistical issues of obtaining and supplying stem cells for therapy. At the same time the functional equivalence of ESCs and iPSCs for experimental, therapeutic, or diagnostic purposes remains questioned, since noticeable differences in gene expression and methylation profiles have been reported along with a considerably higher heterogeneity of iPSCs [5]. The potential candidates for the underlying mechanisms are somatic memory [6], laboratory-specific stochasticity [7], and reprogramming aberrations [8]. Importantly, it was found that reprogramming process manifests deletions of tumor-suppressor genes, and passaging tends to produce duplications of oncogenic genes [9], which poses the question of the stability and clinical safety of iPSCs. Moreover, it was demonstrated that the DNA hypermethylation in cancers preferentially targets the development-associated polycomb group (PcG) proteins and other stemness related loci, and expression patterns of particularly poor differentiated tumors are similar to ESCs, including repression of PcS targets (PCGTs) [10–13]. In this light, identifying markers that would discriminate ESCs and iPSCs and analyzing their potential functional impact, including oncogenetic, appear to be a promising solution.

Considerable advance has been achieved by analyzing variations in methylation profiles of ESCs and iPSCs that evoked dozens of markers, which would account for the differences [14–16]. Furthermore, there is an increasing evidence on the collective nature of such methylation markers, and the first successes due to the large scale machine learning analysis have been reported [17]. These studies, however, concentrated on the variations of methylation levels in separate CpG dinucleotides, which themselves do not characterize the aggregate changes to gene methylation and its coordinated variations in the groups of genes.

Here, led by the results of [13], where intragene methylation measures were introduced to efficiently discriminate cancerous and normal samples by machine learning techniques, we explore their potential as descriptors for EPCs/iPSCs differentiation. We access applicability of the well-established regularized linear and random forest models to confirm their performance. We implement feature selection and analyze the derived sets of top-rank genes for the ESCs/iPSCs for enrichment by the stemness genes and the top cancer gene methylation markers [13]. Altogether, it provides a consistent approach to uncover coordinated variations in the gene methylation profiles between embryonic and induced pluripotent stem cells and quantify similarity of the found best discriminators to the other sets of the known or hypothesized functionality, aiding the quality assessment of reprogramming.

## 2. Materials and Methods

### 2.1. DNA Methylation Data and Descriptors

We analyze genome-wide DNA methylation data collected via the Illumina Infinium Human Methylation 450 BeadChip [14] and available at the NCBI GEO database under the accession designation GSE30654. They contain DNA methylation levels at >450,000 CpG sites, mapped on 18,272 genes for 31 ESCs and 35 iPSCs samples.

A vast number of methylation values as potential features render extremely high-dimensional spaces for machine learning algorithms, additionally complicated by a relatively small number of available samples. Another difficulty is the biological interpretation of a single CpG site methylation importance in distinguishing between different cell types. To overcome these difficulties we propose to describe methylation patterns on a gene level. Following [13], we implement mean (MEAN), variance (VAR), and mean derivative (DERIV) measures, which have proved to be valid in cancer/norm discrimination tasks. In addition, we introduce deviation from a linear pattern (DEV) and asymmetry (ASYMM) measures. The raw methylation values *β* are arranged as they appear along the DNA strand and identify the probes *β*_1_,…, *β*_*n*(*g*)_ located in the region of a specific gene *g*. Denoting the chromosomal coordinate of the corresponding CpG site as coord(*β*_*i*_) (MAPINFO value in terminology of Illumina [18]), we define the gene methylation measures as follows:

(1) MEANg=1ng∑i=1ngβi,VARg=1ng−1∑i=1ngβi−MEANg2,DERIVg=1ng−1∑i=1ng−1βi−βi+1,DEVg=∑i=1ngβ1+βng−β1coordβng−coordβ1coordβi−coordβ1−βi,ASYMMg=∑i=1ng−1βi−βi+1Iβi>βi+1∑i=1ng−1βi−βi+1.

It is worth noting that most of these features require more than one probe per gene. To make sure that all of them are defined and to add more stability to their values we only consider those genes that have ≥5 CpG sites.

**Figure 1:**
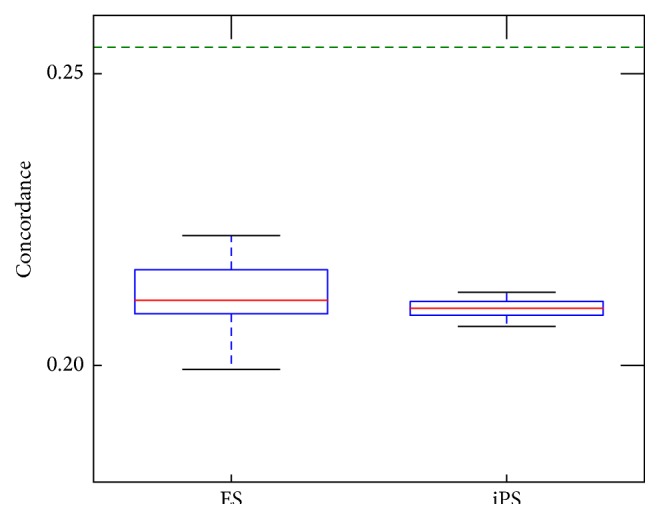
Kendall's coefficient of concordance for the 5 gene-level methylation features summarized over all ES and iPS cells. Green line determines the critical value for the significance level of 5%.

To do a sanity check that these features can complement each other, we compute Kendall's coefficient of concordance based on every single DNA sample and summarize statistics over the whole dataset at hand. As long as the number of features per DNA is fixed we can derive a common critical value for a given significance level (e.g., 5%). Having Kendall's statistics value over this threshold gives a strong argument for the rejection of features disagreement hypothesis.  Figure 1 confirms that features cannot be considered as redundant, and below we explore to what extent they can complement each other.

This diversity of gene-scale aggregate features is intended to capture coordinated variations of intragene methylation profiles across the cell types. Overall, it reduces the dimensionality of the problem and potentially simplifies interpretation of significant descriptors.

### 2.2. Machine Learning Techniques

To solve classification problem we implement the well-established machine learning approaches, regularized linear models, and decision trees. Furthermore, appropriate feature selection routines are employed.

Linear models separate the objects of different classes by a linear decision surface in the feature space. In machine learning, they are known to perform well, when the number of features is much greater than the number of examples, which justifies the choice. In particular, setting a constraint on the linear model parameters via the regularization term in the optimization objective, one can prevent divergence, making the model more stable and relevant, or even make the optimal parameters vector sparse, naturally leading to feature selection. This is realized by logistic regression [19] with *L*_1_ regularizer.

Decision tree is a data mining model with the stepwise decision making procedure. Its nodes are binary decision points assigned with a certain test, which probes for the presence of a particular simple pattern in the data item. Depending on the outcome, one goes to one of the descendant nodes. The procedure is repeated recursively until a leaf is reached and the final decision is taken. In practice, trees are grouped in ensembles to improve the stability of the process. Here, decision trees are trained with the CART algorithm [20], and grouping is realized with the random forest method [21].

Feature importance can be estimated from the set of tests that determine informativity of decision trees in terms of separation of classes [21]. However, as the method itself only ranks features by a certain importance quantifier rather than picking up the meaningful ones, one would have to choose a selection cutoff in the list. A threshold in the importance value would suffer certain arbitrary and, even worse, would lack a transparent relation to the performance.

**Figure 2:**
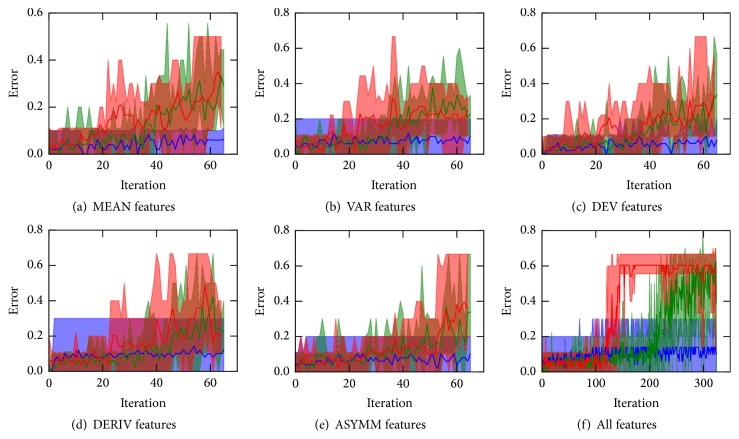
Classification errors of the logistic regression classifiers built on the subsets of features derived by the RBFE procedure. The colored curves show errors of the classifiers utilizing the set of features, selected up to the current iteration (blue), the currently best subset (green), and the remaining features (red). Errors are averaged over the cross-validation folds; the range of the errors is shown in light colors. During feature selection their number was fixed at about 250 and varied during classification to achieve better accuracy.

**Figure 3:**
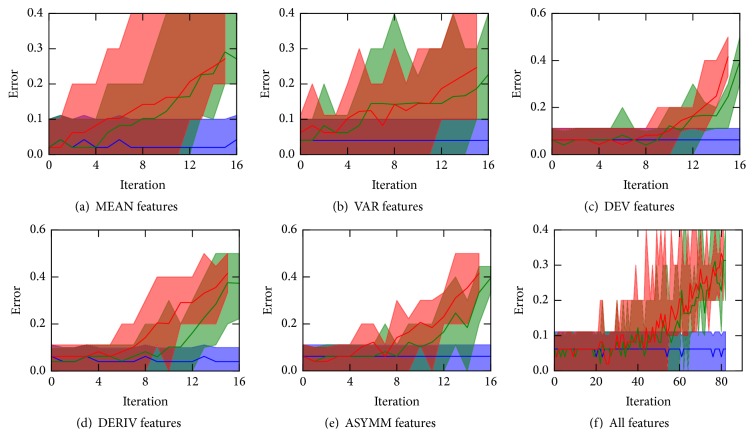
Classification errors of the random forest classifiers built on the subsets of features derived by the RBFE procedure. The colored curves show errors of the classifiers utilizing the set of features, selected up to the current iteration (blue), the currently best subset (green), and the remaining features (red). Errors are averaged over the cross-validation folds; the range of the errors is shown in light colors. A thousand of top-ranked features are selected as the best ones at each iteration.

To overcome these drawbacks, we employ the recursive best feature elimination (RBFE) procedure. The process is started with training the random forest and ranking with the full set of features. In the next step a predefined number of features with top ranks are excluded from the further examination. The procedure is repeated recursively until the remaining feature list becomes empty. In each step we calculate the error of classifiers trained on (i) all selected features, (ii) features selected on the current iteration only, and (iii) the remaining features. In the result, the selection cutoff is informed by the classifier performance curve: one chooses the maximal acceptable classification error for the problem at hand and stops feature selection procedure as the error on current best subset (green curve in Figures 2 and 3) exceeds this threshold.

As the classifier quality is in the heart of feature selection, it is crucial to organize the data flow correctly. We employ the nested 5-fold stratified cross-validation scheme [22]. In case of logistic regression the nested cross-validation is used to calibrate the regularization strength based on the classification accuracy. Hyperparameters, such as regularization coefficient, are tuned in the inner loop, while the outer one estimates the quality of a model. On each fold the subsets of the same cardinality are selected, which makes the averaging more consistent and less noisy.

Binary classification performance (ESCs/iPSCs) is characterized by the three kinds of error: (i) type I error (the fraction of iPSCs erroneously classified as ESCs), (ii) type II error (fraction of ESCs erroneously classified as iPSCs), and (iii) misclassification error (the total fraction of erroneously classified samples).

Data preprocessing is implemented in R programming language [23] and employs Bioconductor [24] packages. Implementation of the machine learning methods is based on the Scikit-Learn library [25] in Python.

### 2.3. Gene Set Enrichment Analysis

To probe for potential aberrations in cell stemness, we assess the enrichment [26] of the best classifiers set by PCGT genes, MESC (Methylated in Embryonic Stem Cells) genes, and PCGT repressed methylated gene groups H3K4, H3K27, and their union (bivalent group) [27], downloaded from the Broad Institute Molecular Signatures Database (http://www.broadinstitute.org/). Searching for the potential cancer-related modifications, we test the enrichment by the 100 genes most significant for discriminating 13 types of tumor [13, Table S1]. These data sets are listed in the Supplementary file S1 in Supplementary Material available online at http://dx.doi.org/10.1155/2015/976362.

## 3. Results and Discussion

### 3.1. ESCs versus iPSCs Classification

First, we explore the potential of logistic regression [19] with *L*_1_ regularizer and random forest [21] for ESCs versus iPSCs classification. As described in Materials and Methods, we make use of the 5-fold cross-validation to estimate the quality of classification. In case of logistic regression we employ nested cross-validation to calibrate the regularization strength based on the classification accuracy. For random forest we always build 1000 trees of depth 3.

Table 1 summarizes performance of these types of classifiers based on different intragene methylation level measures. It shows the average errors of three types along with the standard errors of these estimates (in parenthesis), obtained by cross-validation. Remarkably, both types of classifiers and all intragene measures demonstrate a very good performance with the average accuracy above 95%, improving the previous 90% result, based on the artificial neural network and support vector machine implementations for CpG sites methylation data [17]. Instructively, all the considered intragene features perform in a very similar way with respect to classification quality, which could be understood as multimodal changes in gene methylation due to reprogramming. Substantial standard deviations in error estimates, observed in almost all cases, are likely to be caused by a limited number of samples. It should be noted, however, that the apparently marked differences in the intragene methylation profiles of ES and iPS cells that allow for their confident discrimination do not necessarily imply dramatic functional distinctions, and their biological impact remains to be elucidated.

### 3.2. Feature Selection for ESCs versus iPSCs Classification

Let us proceed with the task of identifying the particular groups of genes, which display significant differences in the intragene methylation profiles between the cell types. In general, a good performance of a classifier only reveals that all intragene methylation features (taken separately or together) contain substantial dissimilarities. To determine a relatively small group of genes that still serves a good discriminator, one has to employ feature selection techniques.

Here we make use of the recursive best feature elimination (RBFE) procedure (cf. Materials and Methods) for both the logistic regression and random forest base classifiers to extract subgroups of intragene features, which nevertheless contain enough information to distinguish between ESCs and iPSCs without compromising the quality.

In fact, *L*_1_ regularized logistic regression model inherently performs feature selection. However, the inspection showed that the results are very unstable, as reflected in the huge variation of subsets cardinality among the cross-validation folds and small intersection of these subsets. It can be a consequence of the limited data volume or an evidence that reprogramming of the cell affects methylation of many genes.

We find that the RBFE procedure performs in a considerably more stable way, as it allows for exploring the features beyond the best ones in terms of the logistic regression. That is, exclusion of the best performing features from the pool shows whether they are crucial for the good classification quality or there are other subsets that perform nearly as well. At each iteration we evaluate three classifiers: (i) utilizing the set of features, selected up to the current iteration, (ii) taking the currently best subset, and (iii) based on the remaining features. During feature selection under the logistic regression model the number of features remains fixed, while the rest of hyperparameters are tuned. The numerical results are reported in Figure 2.

Despite the fact that error curves appear noisy, most probably due to the limited number of samples, it can be clearly seen that the quality of classifiers trained on the currently best and remaining features decreases over RBFE iterations. At the same time, in the initial steps, the decay is negligible if present at all. This observation corroborates the hypothesis that there exist multiple gene subsets approximately equal in terms of their discriminating ability.

A word of caution to be said, regularized linear models are known to lack sufficient power in capturing complex nonlinear relations between the features and the class types. Therefore, it might occur that the features, left out by the RBFE procedure as failing to produce the high-quality linear model, are valid as an input to a nonlinear model.

Random forests, as powerful nonlinear classifiers, usually do not require fine tuning of hyperparameters. As a side advantage, they allow for avoiding the nested cross-validation, leaving only the outer loop for the quality estimation model. However, it should be pointed out that, examining the way the features are utilized in the random forest, one can only rank the features from the most relevant to the totally irrelevant ones. In the RBFE procedure we were selecting 1000 top-ranked features at each iteration. The resulting average cross-validation misclassification error and the range of the scores for all folds are shown in Figure 3.

Overall, the results for logistic regression and random forest models have been proven to be quite similar. For each intragene measure one can identify a subset of features which contain highly important genes, though the size of this subset would still be too large for manual inspection.

### 3.3. Gene Enrichment Analysis

Identification of biological functions associated with the top-ranked classifier genes can be performed by the standard gene enrichment analysis [26]. The method determines whether the overlap between a set of genes of a known or hypothesized functionality and a set of best classifier genes can be explained as a random event. The negative answer implies that a statistically meaningful part of a functional gene group has substantially different methylation profiles in the distinguished cell types. For a quantitative test, we calculate the probability of the null hypothesis, *p* value, that the genes from the functional group found among the best classifiers have entered this set by a random choice from the whole pool of genes. The null hypothesis is rejected if *p* < 0.01.

To probe for potential aberrations in cell stemness, we assess the enrichment of the best classifiers set by PCGT genes, MESC (Methylated in Embryonic Stem Cells) genes, and PCGT repressed methylated gene groups H3K4, H3K27, and their union (bivalent group) [27]. Searching for the potential cancer-related modifications, we test the enrichment by the 100 genes significant for discriminating 13 types of tumor [13, Table S1].

To begin with, we perform feature selection based on decision tree classifiers of depth 1, where cell discrimination is based on a single feature. Sacrificing the accuracy of classification, one benefits from the transparent meaning of the feature importance, which is simply the average misclassification error. For each intragene methylation measure, we arrange genes according to the single gene classifier performance in descending order and consider only those with average error <10%. Then we select a progressively increasing group of best classifier genes, contained between gene #1 and a current gene, and calculate *p* value for the enrichment hypothesis.

**Figure 4:**
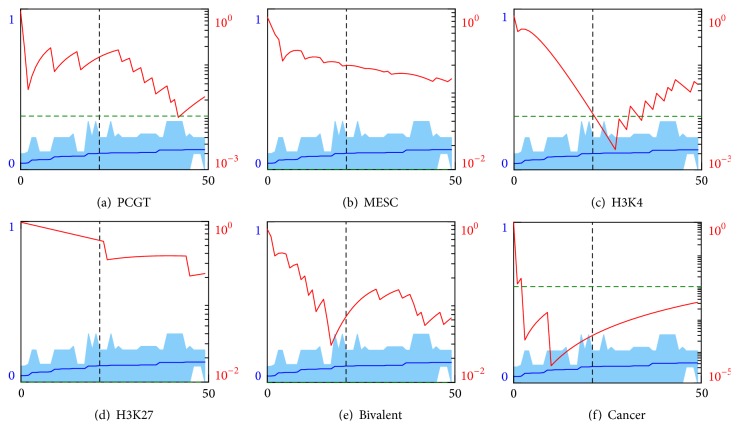
Enrichment analysis of the genes ranked by the misclassification error from the decision tree depth 1 classifier built on MEAN features and sorted in descending order. Horizontal scales indicate gene numbers. Left vertical scales indicate average errors (blue lines) and standard deviations (light blue areas). Black vertical dashed lines indicate the error level 10%. Right vertical scales and red curves indicate *p* values for the enrichment of the best classifiers group, contained between gene #1 and a current gene, by (a) PCGT, (b) MESC, (c) H3K4, (d) H3K27, (e) bivalent, and (f) cancer gene groups. Green dashed lines indicate the null hypothesis rejection threshold of *p* = 0.01 probability.

**Figure 5:**
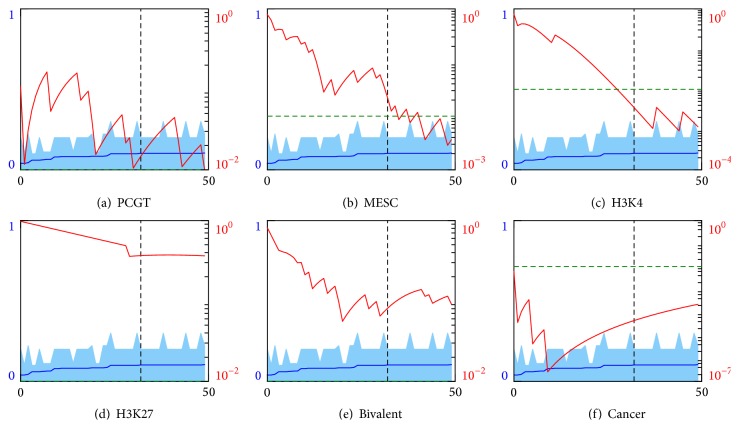
Enrichment analysis of the genes ranked by the misclassification error from the decision tree depth 1 classifier built on VAR features and sorted in descending order. Horizontal scales indicate gene numbers. Left vertical scales indicate average errors (blue lines) and standard deviations (light blue areas). Black vertical dashed lines indicate the error level 10%. Right vertical scales and red curves indicate *p* values for the enrichment of the best classifiers group, contained between gene #1 and a current gene, by (a) PCGT, (b) MESC, (c) H3K4, (d) H3K27, (e) bivalent, and (f) cancer gene groups. Green dashed lines indicate the null hypothesis rejection threshold of *p* = 0.01 probability.

The results for MEAN and VAR measures are shown in Figures 4 and 5, respectively. (The other measures do not manifest significant enrichment and the corresponding plots are not shown.) Remarkably, we find that the hypothesis of enrichment by any of the stemness-related functional groups does not pass the accepted *p* = 0.01 rejection threshold. Quite on the contrary both MEAN and VAR measure based best classifiers groups do exhibit a statistically meaningful enrichment by the cancer discriminating genes.

**Figure 6:**
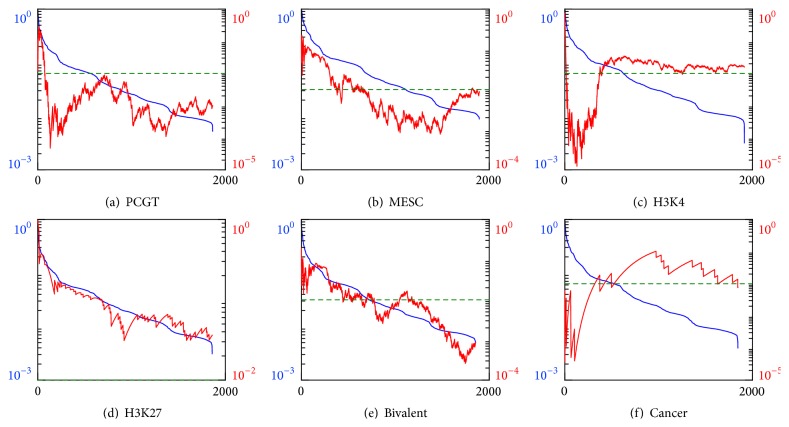
Enrichment analysis of the genes that are top-ranked by the random forest classifier built on the MEAN features and sorted according to their importance scores (blue lines and left vertical scales) in descending order. Red curves and right vertical scales indicate *p* values for the enrichment of the best classifiers group, contained between gene #1 and a current gene, by (a) PCGT, (b) MESC, (c) H3K4, (d) H3K27, (e) bivalent, and (f) cancer gene groups. Horizontal scales indicate gene numbers. Green dashed line indicates the null hypothesis rejection threshold of 1% probability.

**Figure 7:**
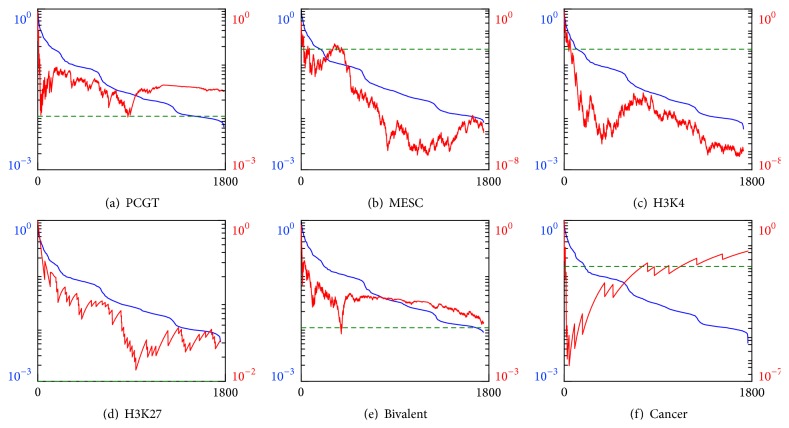
Enrichment analysis of the genes that are top-ranked by the random forest classifier built on the VAR features and sorted according to their importance scores (blue lines and left vertical scales) in descending order. Red curves and right vertical scales indicate *p* values for the enrichment of the best classifiers group, contained between gene #1 and a current gene, by (a) PCGT, (b) MESC, (c) H3K4, (d) H3K27, (e) bivalent, and (f) cancer gene groups. Horizontal scales indicate gene numbers. Green dashed line indicates the null hypothesis rejection threshold of 1% probability.

Repeating the procedure for random forest classifiers we not only confirm the pronounced enrichment by cancer markers but also uncover significant enrichment by PCGT (MEAN measure), MESC, and H3K4 (MEAN and VAR measures) gene groups; see Figures 6 and 7. This result can be explained by a better performance and sensitivity of multifeature classifiers. Presumably, they uncover hidden variations in the iPSCs methylation profiles, coordinated between gene subsets and missed by the trivial single-feature classifiers.

Finally, we note that while the meaningful differences in the stemness and cancerous gene groups between the ES an iPS cell states are identified, the conclusions on the equivalence of iPSCs and ESCs and safety of iPSCs cannot be made straightforwardly. Whether the distinction in methylation patterns leads to the biologically pronounced effects has to be verified separately. Here, our analysis provides a good starting point.

## 4. Conclusions

We studied the potential of differentiating embryonic and induced pluripotent stem cells by the regularized linear and decision tree machine learning classification algorithms, based on a number of intragene methylation measures. The resulting average accuracy of classification has been proven to be above 95%, which overcomes the earlier achievements. We proposed a constructive and transparent method of feature selection based on classifier accuracy instead of the feature importance. Enrichment analysis revealed statistically meaningful presence of stemness group and cancer discriminating genes among the selected best classifying features. These findings stimulate the further studies to determine the functional consequences of these differences in methylation patterns. The presented approach can be broadly applied to discriminate the cells of different phenotype or in different state by their methylation profiles, identify groups of genes constituting multifeature classifiers, and assess enrichment of these groups by the sets of genes with a functionality of interest.

## Supplementary Material

Table S1. List of genes used to test the enrichment: PCGT and MESC genes, PCGT repressed methylated gene groups H3H4, H3K27 and their union (bivalent group), and 100 mostly unstable genes for discrimination of 13 types of cancer [13].

## Figures and Tables

**Table 1:** ESCs versus iPSCs classification performance for linear regression and random forest based algorithms and for different types of intragene methylation measures, taken separately or together. Standard deviations of the estimates are given in parenthesis.

Features type	Type I error	Type II error	Misclassification error
*Logistic regression*			

MEAN	0.12 (±0.15)	0.00 (±0.00)	0.04 (±0.05)
VAR	0.05 (±0.10)	0.07 (±0.08)	0.06 (±0.05)
DEV	0.00 (±0.00)	0.07 (±0.08)	0.04 (±0.05)
DERIV	0.00 (±0.00)	0.03 (±0.07)	0.02 (±0.04)
ASYMM	0.00 (±0.00)	0.03 (±0.07)	0.02 (±0.04)
All features	0.00 (±0.00)	0.03 (±0.07)	0.02 (±0.04)

*Random forest*			

MEAN	0.00 (±0.00)	0.03 (±0.07)	0.02 (±0.04)
VAR	0.00 (±0.00)	0.07 (±0.08)	0.04 (±0.05)
DEV	0.07 (±0.13)	0.07 (±0.08)	0.06 (±0.05)
DERIV	0.00 (±0.00)	0.07 (±0.08)	0.04 (±0.05)
ASYMM	0.07 (±0.13)	0.07 (±0.08)	0.06 (±0.05)
All features	0.00 (±0.00)	0.07 (±0.08)	0.04 (±0.05)
